# Dopant morphology as the factor limiting graphene conductivity

**DOI:** 10.1038/srep17393

**Published:** 2015-11-30

**Authors:** Mario Hofmann, Ya-Ping Hsieh, Kai-Wen Chang, He-Guang Tsai, Tzung-Te Chen

**Affiliations:** 1 Department of Material Science and Engineering, National Cheng Kung University, Tainan 70101, Taiwan; 2 Graduate Institute of Opto-Mechatronics, National Chung Cheng University, 168 University Road, Min-Hsiung Township, Chiayi County 62102, Taiwan; 3 Electronics and Optoelectronics Research Laboratories, Industrial Technology Research Institute, Chutung, Hsinchu 31040, Taiwan, ROC

## Abstract

Graphene’s low intrinsic carrier concentration necessitates extrinsic doping to enhance its conductivity and improve its performance for application as electrodes or transparent conductors. Despite this importance limited knowledge of the doping process at application-relevant conditions exists. Employing *in-situ* carrier transport and Raman characterization of different dopants, we here explore the fundamental mechanisms limiting the effectiveness of doping at different doping levels. Three distinct transport regimes for increasing dopant concentration could be identified. First the agglomeration of dopants into clusters provides a route to increase the graphene conductivity through formation of ordered scatterers. As the cluster grows, the charge transfer efficiency between graphene and additional dopants decreases due to emerging polarization effects. Finally, large dopant clusters hinder the carrier motion and cause percolative transport that leads to an unexpected change of the Hall effect. The presented results help identifying the range of beneficial doping density and guide the choice of suitable dopants for graphene’s future applications.

Graphene, a two-dimensional carbon system, is considered an enabling material in many fields. One of the earliest commercial uses could be as a transparent conducting film for touch screens or display applications or as electrodes in energy storage solutions^1^.

Enhanced performance of these devices requires the reduction of graphene’s electrical resistance. The most common approach for this task is to increase graphene’s intrinsically low carrier density through extrinsic doping^2^. A variety of materials can be used to increase the carrier density of graphene through charge transfer processes, including liquids, polymers, metals and gasses^3^.

Despite significant efforts, the enhancement in carrier concentration by doping is limited and with it the sheet resistance reduction^3^,^4^. This issue arises from the incomplete understanding of the doping process. Theoretical studies of charge transfer are carried out at the individual dopant level^5^ and experiments are conducted in the low dopant density regime^6^. Extrapolation from these experiments towards the application relevant doping region seems problematic and currently the search for suitable dopants proceeds often through trial-and-error^4^. Future optimization strategies require insight into what properties are relevant for an effective graphene dopant and what range of doping is achievable.

We here present an *in-situ* study of the effect of doping on the transport properties of graphene. A measurement system was employed that combines sheet resistance, Hall effect, and Raman measurements (Fig. 1(a)) which allows identifying the interaction between graphene and various dopants (AuCl_3_, HNO_3_, and ozone) and its correlation with carrier transport.

Three distinct transport regimes could be identified for these dopants: At low dopant concentration, the agglomeration of dopants into clusters provides a route to increase the graphene conductivity through formation of ordered scatterers. As these clusters grow, the charge transfer efficiency from dopants to graphene decreases due to developing electric fields. Finally, at high dopant concentration, large dopant clusters hinder the carrier motion and cause percolative transport. These findings reveal the importance of the dopant morphology as factors benefitting and limiting the carrier transport in two-dimensional materials.

## Methods

Graphene was synthesized by chemical vapor deposition on Cu foil following previous reports^7^. Briefly, Cu-foil (Alfa Aesar 13382) was pretreated using electropolishing and annealing at 1000C for 30 minutes under an atmosphere of Hydrogen. Graphene growth was conducted in a quartz tube at 1000 °C using CH_4_ at a pressure of 100 mTorr. Thus grown graphene was then transferred onto SiO_2_ samples^8^ and contacted using conductive silver adhesive.

**Figure 1:**
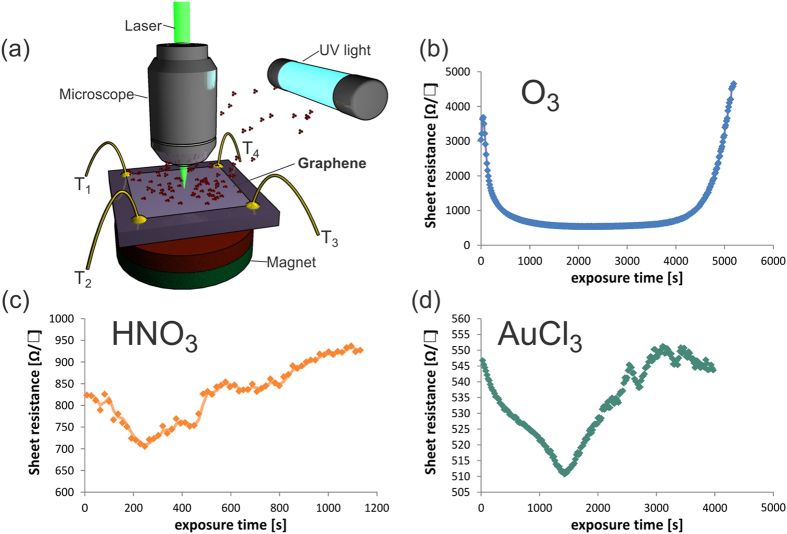
(**a**) Schematic of measurement setup for Ozone doping experiments, (**b–d**) sheet resistance vs. time for (**b**) AuCl_3_, (c) HNO_3_, (**d**) ozone doping.

The samples were attached to a home-made measurement setup (Fig. 1(a)): A LabView controlled Agilent B2900A source meter was employed to continuously measure the graphene sheet resistance in 4 probe van-der-Pauw configuration over time. Using a computer controlled switching matrix, the same contacts were used to measure the Hall voltage and calculate Hall-effect mobility. A magnetic field of 0.5T was provided by a permanent magnet positioned underneath the sample. Raman spectroscopy was conducted simultaneously using a homebuilt system with a 514 nm light source.

Three different dopants were investigated, i.e. UV generated ozone, AuCl_3_, and HNO_3_.

Ozone was generated through dissociation of air by UV irradiation. For this purpose, a light source with emission at 185 nm and 254 nm was positioned in the vicinity of the sample (Fig. 1(a)).

Continuous AuCl_3_ doping was achieved by placing the sample holder upside-down onto a vial of 0.1M aqueous AuCl_3_ solution. After the measurement was started, the vial was heated to 80 °C to initiate evaporation of the solution. Condensation of the solution on the sample resulted in a continuous increase of AuCl_3_ coverage with time. Continuous HNO_3_ doping was achieved with the same approach using a 0.25M solution of HNO_3_ in water.

## Results and Discussion

Time-resolved measurements were carried out during the exposure of graphene to different dopants while simultaneously monitoring Raman spectra, sheet resistance, Hall mobility, and carrier concentration. Figure 1(b) shows the change of graphene sheet resistance upon interaction with ozone. Three distinct regimes can be identified from this figure: For short exposure times the sheet resistance decreases until it stagnates at a low value. In an intermediate period, no change in sheet resistance occurs. Finally, after long exposure duration the sheet resistance increases significantly. This behavior is very similar to the evolution of sheet resistance upon HNO_3_ and AuCl_3_ vapor exposure (Fig. 1(c,d), respectively), albeit at different scales. Here, too, the sheet resistances decrease until they reach a minimum and then increase with time. The similarity of graphene’s behavior for exposure to different dopants suggests a universal relation between dopant and carrier transport. These results also indicate that an optimum doping density exists and if one ignores this “sweet spot” a suboptimal performance is attained. Only through understanding this underlying mechanism the optimum dopant concentration and the limits of graphene’s achievable performance can be identified.

Theoretical descriptions of carrier transport in graphene in the presence of dopants have focused on two mechanisms, Coulomb scattering and short-range scattering^9^,^10^. Both scattering mechanisms result in mobilities that are inversely proportional to the impurity concentration^11^


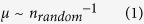


**Figure 2:**
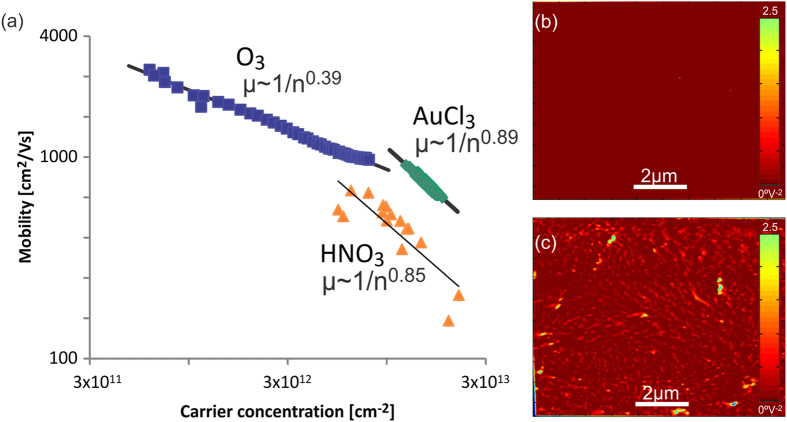
Cluster formation: (**a**) log-log-plot of carrier mobility vs. carrier density for different dopants, (**b**) EFM before ozone exposure, (**c**) EFM after short exposure on same color scale.

Figure 2(a) shows a plot of mobility vs. carrier density for short dopant exposures on a log-log scale. Assuming that the carrier concentration is proportional to the impurity concentration *n*~*n*_*random*_ and using the above description, a slope of −1 would be expected for both Coulombic scatterers and short range scatterers in such a plot. Instead, we observe slopes smaller than −1 for all three dopants. This behavior indicates that the mobility is less affected by an increase in scatterer concentration than the carrier density.


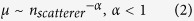


Such a behavior was previously found for the interaction of Au-clusters with pristine graphene and attributed to cluster formation which cause a smaller scattering cross section than random spatial distributions^12^. We therefore conclude that at the investigated doping conditions “ordered scatterers” are dominating the carrier transport. Consequently, the achievable sheet resistance decrease is determined by the ratio of scattering cross section and charge donation ability expressed by *α*


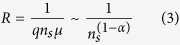


Cluster formation has been observed experimentally for AuCl_3_ doping^8^ but was only theoretically predicted for ozone^13^. To confirm the arrangement of ozone in charged clusters we carried out electrostatic force microscopy (EFM). In this technique, effects of charge accumulation and work function differences are inferred from changes in the phase of a biased oscillating cantilever. Since such phase changes are also associated with differences in surface properties^14^, EFM measurements are traditionally cumbersome to interpret. We employ a novel analysis technique by Lilliu *et al.* who analyzed the phase change as a function of cantilever bias for each pixel of the investigated sample area^15^. The extracted information is proportional to the contact potential difference due to changes in the work function and Fig. 2(b,c) show the acquired data across graphene samples before and after exposure to UV-ozone (more information is provided in the Supporting information). Formation of clusters can clearly be seen which corroborates our previous hypothesis. Analysis reveals an average ozone cluster diameter of 100 nm which is significantly larger than the dimensions of Au cluster (20 nm)^8^. This difference in cluster size could explain the smaller *α* of Ozone observed in Fig. 2(a), since the scattering cross section is expected to scale with the cluster concentration^16^. More detailed theoretical work is necessary to quantify the impact of cluster size on *α* and extract the impact of materials parameters on their properties as ordered scatterers.

The simple relation between sheet resistance and doping in Eq. (3) suggests that the sheet resistance can be decreased indefinitely as long as the dopant concentration can be increased. This model is at odds with our observation that the sheet resistance instead increases for long exposure (Fig. 1(b–d)). Therefore another mechanism has to exist that increases the sheet resistance for higher doping concentration.

In the case of ozone doping, the formation of lattice defects for long exposures at high ozone concentration had been put forward as a possible competing mechanism^17^. In our experiments, however, the concentration of ozone is small and Raman analysis does not reveal amorphization (Suppl Figure S1(b)). Furthermore, the increase in sheet resistance in the high doping regime can be completely reversed by mild heating to 100 °C (Figure S1(a)). We therefore conclude that the increase in sheet resistance is only due to effects of dopant adsorption and no lattice defect formation occurs. This conclusion can also explain the similarities in behavior with other dopants that are not known to induce lattice defects.

**Figure 3:**
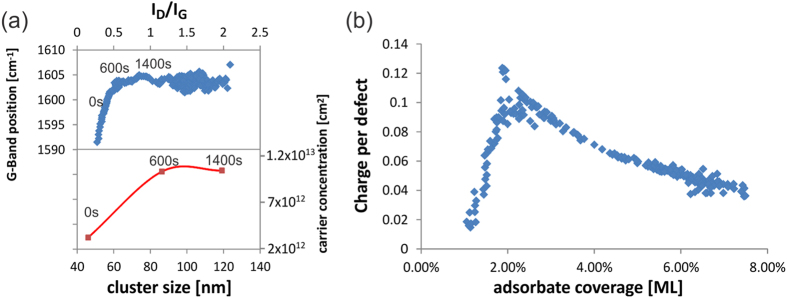
Characterization of ozone adsorption: (**a**) (top) time evolution of Raman G-band position vs. I_D_/I_G_ ratio (bottom) time evolution of adsorbate cluster dimension from EFM vs. according carrier concentration, (**b**) charge per adsorbate for increasing coverage.

We characterize the effect of adsorbates on graphene’s transport properties by analyzing the defect related Raman I_D_/I_G_ ratio and the G-Band position during ozone exposure (Fig. 3(a)). The I_D_/I_G_ ratio is proportional to the adsorbate density (see Supporting Information for a detailed description) whereas Raman G-Band position increases with carrier density^18^. The observed initial proportionality between the parameters indicates a charge transfer between adsorbates and graphene as expected from doping. At intermediate exposure durations, however, the I_D_/I_G_ ratio increases without a significant change in G-Band shift. This behavior is unexpected since charge transfer should occur as long as adsorption happens. The same behavior is observed when correlating the evolution of dopant morphology from EFM with electrical transport measurements. Figure 3(a) shows that the clusters keep growing even after the carrier concentration reaches equilibrium.

To understand this behavior, we try to estimate the number of charges transferred from each formed adsorbate/graphene bond by comparing the charge carrier concentration from electrical measurements to the concentration of adsorbate extracted by Raman analysis (See Supporting Information for more details). Figure 3(b) shows that the number of charges per defects reaches a maximum of ~0.12 which agrees with previous measurements of the oxygen charge transfer to graphene upon weak chemisorption^19^. This amount of charge transferred is not only much smaller than expected from simulations^5^ but also decreases with increasing adsorbate concentration. Our observation indicates that at high adsorbate concentration, the deposition of more adsorbates does not increase the number of donated charges. Therefore the maximum carrier density is not determined by the density of adsorbates but by limits of the charge transfer efficiency.

The decrease in charge transfer efficiency for increasing adsorbate densities has been studied in the field of the electronic theory of catalysis (ETC) in the late 1950s^20^: Upon interaction with a surface an adsorbate is left (partially) ionized and therefore charged. If a second molecule adsorbs in the vicinity of this charged center it experiences an electrical field which decreases its own ionization efficiency. Consequently, charge transfer is more efficient for individual adsorbates than for clusters. Klier *et al.* modeled this situation in three dimensions and predicted a decrease in the number of transferred charges N per number of adsorbate n_0_^21^. The transfer efficiency *N*/*n*_0_ was predicted to scale with 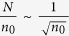 which agrees with the decay observed in Fig. 3(b). An interesting prediction of the ETC theory is the relative independence of the transfer efficiency on the work function of the adsorbate which can explain why various dopants exhibit similar limiting doping levels that are only weakly depending on the dopant work function^4^. (More detailed explanations are provided in the Supporting Information.)

**Figure 4:**
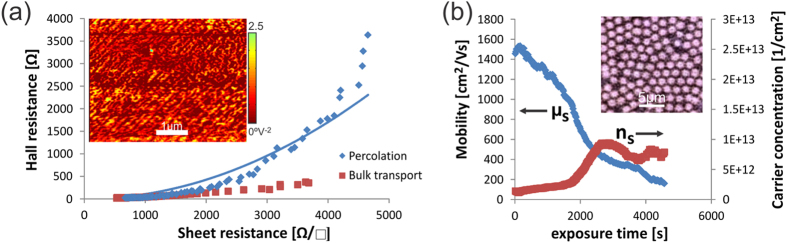
Transport at high coverage (**a**) Hall resistance vs. sheet resistance at low and high coverage (inset) EFM image of adsorbates at high coverage, (**b**) transport after suppression of percolation by formation of adsorbate super lattice, (inset) micrograph of microsphere array.

We now turn to the high doping concentration regime. EFM imaging in this range reveals that clusters have extended far enough to merge with neighboring clusters (inset of Fig. 4(a)). In this case two continuous phases exist – graphene and adsorbate-covered graphene. Due to the previously mentioned electric fields at the interface of these two regions, barriers exist that prevent charge transport between them. Instead, percolative carrier transport will proceed mainly through one region.

Hall effect measurements, that are generally regarded as the gold standard in the measurement of carrier concentration and mobility^22^, exhibit surprising features in the percolative regime. In general, the Hall resistance *ρ*_*H*_ is a robust measure of carrier density even in the presence of disorder, such as grain boundaries, polycrystallinity or random defects^23^. For percolative transport, however, the total Hall resistance is not only dependent on the Hall resistance in the graphene phase 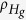 but also on the Hall resistance in the adsorbate covered phase 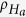 and graphene’s sheet resistance^24^. Consequently, the transition from bulk transport to percolative transport is accompanied by an increasing Hall resistance as shown in Fig. 4(b). Ignoring this effect will result in an underestimation of the carrier density and an overestimation of the mobility. Measurement errors at very high Hall resistances could even be interpreted as a change of carrier type as had been observed previously^17^.

The dependence of Hall effect measurements on the properties of both phases allows us to extract the characteristics of adsorbate-covered graphene. In percolative systems the Hall resistance scales with the sheet resistance and the Hall resistances of the constituting phases^24^.


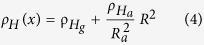


Figure 4(c) shows a fit of our data to Equation 5. Based on the extracted fitting parameters and estimates for the Hall resistances of the two phases, we extrapolate to the resistance of the adsorbate phase of 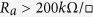. This resistance is several orders of magnitude higher than the resistance of pristine graphene and therefore at complete adsorbate coverage graphene is rendered insulating. This limit of high doping reveals that suitable doping has to be in the low coverage regime and merging of adjacent adsorbate clusters has to be avoided to prevent turning graphene into an insulator even in the absence of lattice defect formation.

One way of accomplishing this goal is to control the position of the adsorbate clusters. We employed microsphere lithography^25^ to introduce large spacing between adsorbate clusters (inset Fig. 4(b)).

Without the effects of percolation influencing the Hall effect measurements, we can see that the mobility decreases continuously as more and more dopants adsorb and clusters grow throughout the experiment. The carrier density, however, reaches saturation as the clusters extend beyond a critical size which corroborates our explanation of the importance of dopant morphology on the limits of doping.

## Conclusion

The presented results reveal several important characteristics of the doping process.

1. Clustering of adsorbates is necessary to cause doping-induced enhancement of the graphene conductivity.
2. Electric fields that develop in growing clusters limit the maximum achievable doping. The achievable limit is only weakly dependent on the nature of the adsorbate.
3. The adsorbate phase is almost insulating and its main role is to provide carriers to the pristine region without taking part in the carrier transport significantly.

Concluding from these observations we identify the dopant cluster dimension as the most important factor for graphene doping. To achieve high mobility, dopants should be concentrated in few large clusters. However, efficient charge transfer from adsorbates to graphene requires many small clusters.

This competition is illustrated when comparing the three different dopants utilized in this study. Ozone has an enhanced sublinearity between dopant density and mobility (Fig. 2(a)) indicating significant correlation of scatterers as found in large clusters. On the other hand the maximum carrier density is low because the occurring large clusters limit charge transfer. AuCl_3_ and HNO_3_ seem to produce more dopant clusters of smaller dimensions which decrease the sublinearity but enhance the charge transfer and maximum charge density. These results are confirmed by AFM imaging of AuCl_3_ clusters at different exposures (Suppl. Figure S6).

We can finally identify the hallmarks of a good dopant: It has to exhibit a high dielectric constant to increase the amount of transferred charge and a low work function to reach stable adsorption at low coverage. Furthermore, the surface free energy has to be high enough to form compact clusters on graphene. Based on these characteristics metal oxides and conjugated polymers should be considered for the efficient doping of graphene for future applications.

## Additional Information

**How to cite this article**: Hofmann, M. *et al.* Dopant morphology as the factor limiting graphene conductivity. *Sci. Rep.* **5**, 17393; doi: 10.1038/srep17393 (2015).

## Supplementary Material

Supplementary Information
