# Supplementary material for: Dopant morphology as the factor limiting graphene conductivity
Source: Sci Rep. 2015 Nov 30;5:17393. doi: 10.1038/srep17393 (PMC4663794; doi:10.1038/srep17393)
Supplement: Supplementary Information [file srep17393-s1.pdf]

Supporting Information

Dopant morphology as the factor limiting graphene conductivity

Mario Hofmann[1], Ya-Ping Hsieh[2], Kai-Wen Chang[1], He-Guang Tsai[2], Tzung-Te Chen[3] and Chia-Chen Hsu[4]

1. Department of Material Science and Engineering, National Cheng Kung University, Tainan, Taiwan 70101
2. Graduate Institute of Opto-Mechatronics, National Chung Cheng University, 168 University Road, Min-Hsiung Township, Chiayi County, Taiwan 62102
3. Electronics and Optoelectronics Research Laboratories, Industrial Technology Research Institute, Chutung, Hsinchu 31040, Taiwan, ROC
4. Department of Physics, National Chung Cheng University, 168 University Road, Min-Hsiung Township, Chiayi County, Taiwan 62102

Reversibility of transport regimes

Figure S1(a) shows the sheet resistance evolution with ozone exposure time for an experiment that was interrupted at various points to introduce mild heating (T<100°C). It can be seen that the graphene can be returned to its initial condition upon which it will traverse the same conductance regimes again. This reversible process suggests that only adsorption of dopants causes all three conductance regimes and graphene does not undergo substantial defect formation itself. (The increase in minimum resistance for subsequent cycles indicates that a small amount of lattice defects could have been introduced through prolonged UV exposure or heating in air).

Furthermore, desorption experiments were carried out in vacuum conditions and a significantly faster desorption was observed. The lower minimum carrier concentration in Figure S1(b) suggests that co-desorption of oxygen from the graphene occurs under these conditions.

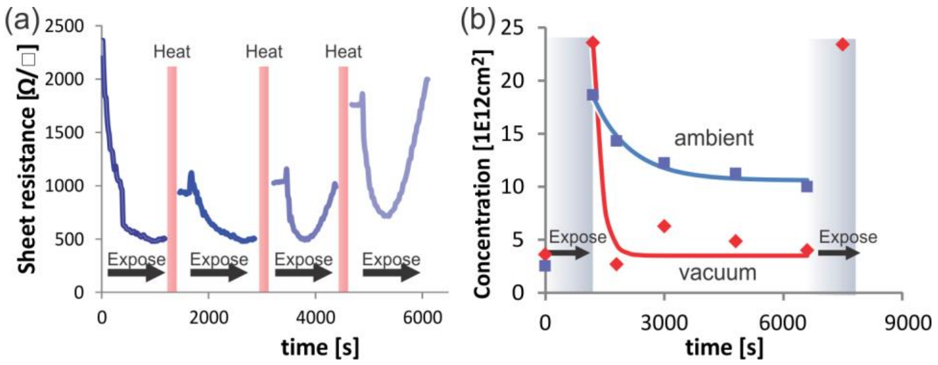

Figure S1. (**a**) Reversible sheet resistance evolution through heating, (**b**) Carrier concentration evolution upon ozone desorption in vacuum and in ambient conditions

Extraction of binding energy

The time evolution of the absorbed molecule coverage Θ can be quantified by a Langmuir model.

$$\frac{d\Theta}{dt} = k_a pN(1 - \Theta) - k_d \mathrm{N}\Theta$$

,where k_a and k_d are the rates of adsorption and desorption, respectively, and p is the partial pressure of ozone.

Upon deactivation of the UV illumination, the ozone partial pressure quickly reaches zero and the coverage follows:

$$\Theta(t) = \Theta_0 exp^{-k_d N t}$$

The coverage can be estimated by the change in carrier concentration (Figure S1(b)) and the binding energy can be extracted from the desorption rate

$$k_d N = \nu_0 \exp(-E_b/kT)$$

Using fits for 12 different desorption experiments and reported values of$\nu_0 = 2 \times 10^{13} s^{-1}$, we obtain a binding energy of .$0.90eV \pm 0.02eV$ which is remarkably close to previously found values for ozone chemisorption ($E_B = 0.8eV$) and much larger than the value for ozone physisorption($E_B = 0.25eV$) [1].

Detailed Raman characterization

Figure S2 (b) shows the evolution of the D-Band to G-Band intensity ratio as a function of exposure time for an uninterrupted exposure experiment. A monotonous increase of the intensity ratio without an inflection point suggests that we are operating in the low defect regime without amorphization occurring[2] which is due to the lower ozone concentration in our experiments compared to previous reports[3].

The question arises, where the increasing I_D/I_G ratio originates from if no lattice defects are formed. To address this issue, we conducted an experiment where the UV light source is switched on and off. We observe that the I_D/I_G ratio decreases over time after the UV light source is switched off (Figure S2(c)). Since the experiment is carried out at room temperature and small laser power, no healing of lattice defects is expected[4]. The only occurring change under dark condition is the decrease in ozone concentration due to desorption and recombination[1].

The similarity between charge concentration and Raman I_D/I_G ratio in Figure S2(c) suggests that Raman characterization is sensitive not only to lattice defects but to adsorbates that cause charge transfer, such as previously suggested epoxy groups[1]. This effect of adsorbates on the I_D/I_G ratio behavior can be explained through occurring charge transfer between functional groups and individual carbon atoms that will locally stiffen individual carbon bonds in the graphene lattice and thus break the symmetry and increase the D-Band intensity even in the absence of graphene lattice defects[5].

These results suggest that graphene's I_D/I_G ratio can be used to monitor the ozone-assisted formation of adsorbates independent of their charge transfer characteristics. Raman and electrical measurements are therefore two complementary tools to measure the effect of dopant

concentration on the carrier transport in the case of ozone adsorption.

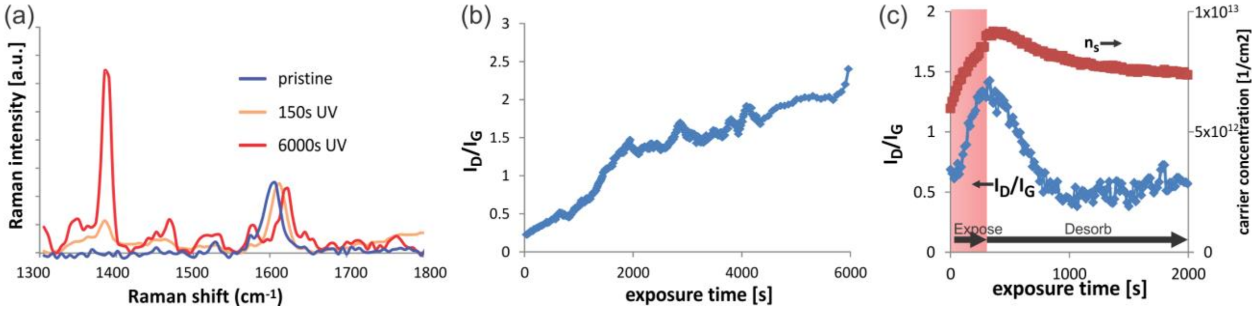

Figure S1. Raman analysis of ozone adsorption: (**a**) Representative Raman spectra after different durations of UV exposure, (**b**) Raman D-Band/G-Band intensity ratio vs. time, (**c**) simultaneous I_D/I_G and carrier concentration evolution under illuminated and dark conditions

Quantifying adsorbate coverage by Raman spectroscopy

We can utilize the observation that ozone coverage can be characterized by Raman spectroscopy to identify the physical mechanism of doping. To accomplish this goal, we attempt to quantify the effect of ozone generated adsorbates on the I_D/I_G ratio. The I_D/I_G ratio has previously been related to the number of defects by an empirical formula[2]. This formula, however, was developed for randomly distributed defects and not clusters. Since clustering of epoxide groups will occur at dimensions that are smaller than the defect exciton radius the contribution of one epoxide group will be overestimated by the formula. That is, cluster of a large number of adsorbates will look like a single defect as long as its dimensions are smaller than the exciton radius. The exciton radius in graphene has been estimated to be approximately 3.1nm[2] while simulations[6] place the energetically epoxide density at $0.5nm^2$.. We therefore estimate that approximately 600 clustered

epoxide groups contribute to one Raman active defect. Consequently the formula by Cancado was modified to:

$$n_{epoxy}(cm^{-2}) = \frac{3\times10^{19}}{\lambda_L^4}\left(\frac{I_D}{I_G}\right)$$

Using this density, we can normalize by the total number of carbon atoms per cm^2. The adsorbate coverage for the different stages of our experiment can now be assessed (Figure S3): For a surface coverage of ~1% a decrease in sheet resistance occurs. For higher coverage the sheet resistance stays constant and if the surface coverage exceeds 6% the resistance increases. The maximum coverage of ~8.5% agrees well with predictions for room temperature epoxide coverage at low ozone concentrations of 10%.[1]

Our finding suggests that only a low adsorbate coverage is beneficial to improving the graphene sheet resistance whereas higher adsorbate concentrations result in an increased resistance due to the onset of percolative transport.

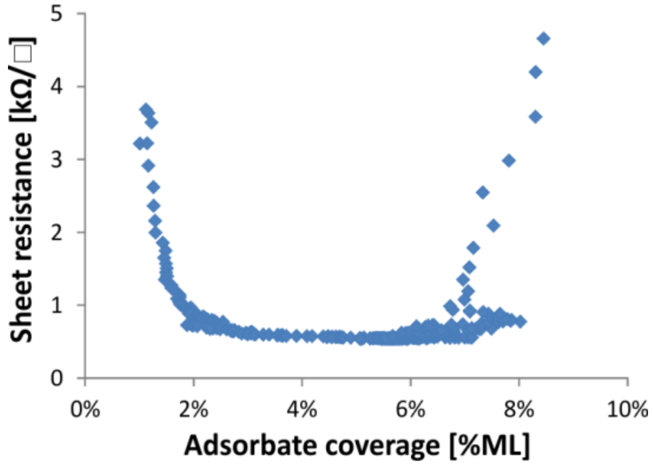

Figure S3. Sheet resistance vs. ozone coverage

EFM measurements

Extraction of work function differences from electrostatic force microscopy was carried out following Lilliu et al.[7]: Lifting phase images of the same sample area were collected at 7 cantilever bias values between -3 to 3V. Image registration was employed to ensure proper alignment of these 7 images. Then, fitting to a quadratic function was performed for each image pixel. The obtained fitting parameters are plotted in image form and represent a change in work function that is independent of sample morphology.

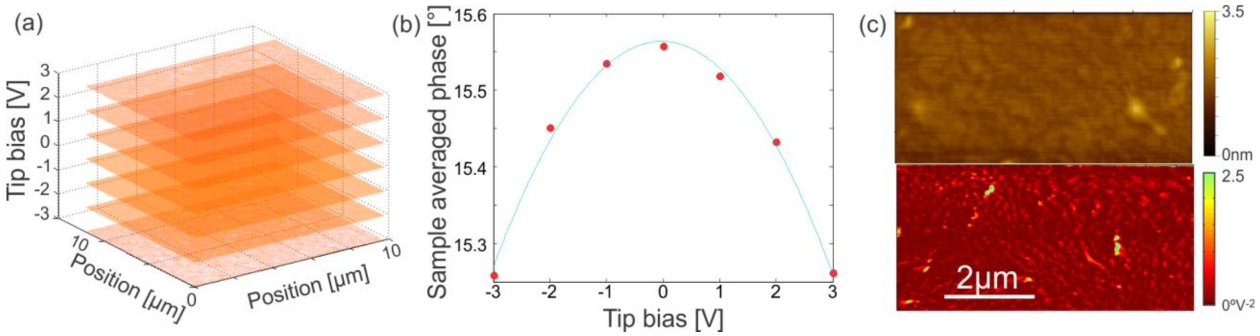

Figure S4. EFM parameter extraction: (**a**) plot of phase lifting images of same area as function of tip bias, (**b**) image averaged phase vs. tip bias with quadratic fit, **(c)** comparison of morphology (top) and EFM data (bottom)

ETC modeling of adsorbate charge transfer

Klier modeled the effect of incomplete ionization of adsorbates due to electrostatic interaction[8] and obtained a relation between the number of adsorbates (n_0) and the amount of charge transferred (N).

$$N = \sqrt{\frac{\varepsilon kT}{2\pi e^2}\left(1 - \exp(-\frac{e(V_0 - V_S)}{kT})\right) n_0} \qquad (5)$$

Here ε is the dielectric constant of the cluster and $V_0 - V_S$ is the difference in potential between center and boundary of the cluster.

Impact of work function on charge transfer efficiency

Based on this model, we can evaluate the effects of different materials on doping: At complete depletion of the cluster, the potential difference between cluster boundary and center is equal to the work function difference between graphene and the dopant. This parameter, however, is only weakly affecting the achievable transferred charge N which can explain why graphene doping seems to only weakly depend on the nature of the adsorbate[9]. Instead we find that the cluster dimension is a critical factor in determining the character of doping and that a large cluster has a lower efficiency of charge transfer.

The competition of this effect with the previously observed enhancement of mobility for large dopant clusters cause a size region where a cluster's advantage in mobility is canceled out by the detriment in charge transfer. In such a regime the sheet resistance is not improving due to additional doping as seen in the coverage regime between 3% and 6% of Figure S2. This constant resistance region is remarkably stable over a wide range of adsorbate coverage. When relying solely on electrical measurements this behavior could be misinterpreted as complete adsorption[10] or equilibration[11]. Raman spectroscopy, however, reveals that the adsorbate concentration keeps increasing even after the doping induced shift of the G-band has reached equilibrium (Figure S5).

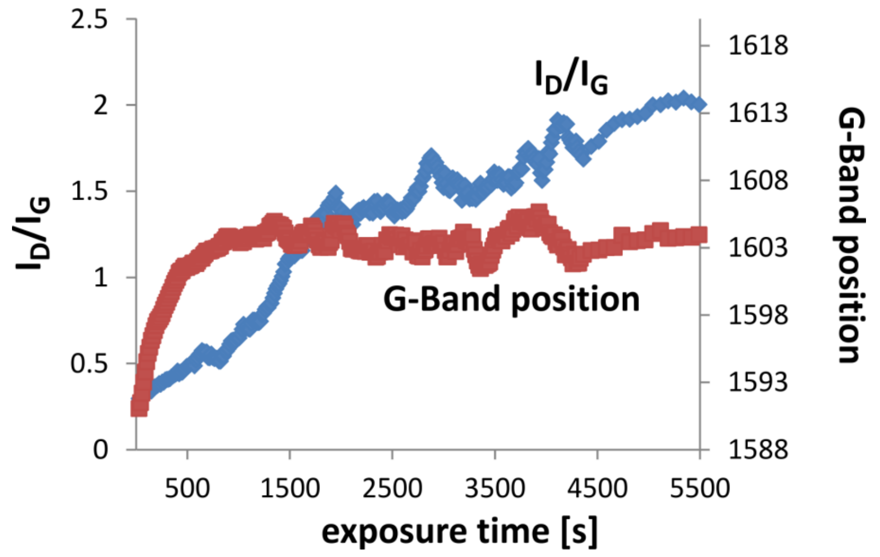

Figure S5. Raman G-Band position and ID/IG ratio vs. exposure time

Extraction of adsorbate-covered graphene resistance

$$R_a = \sqrt{\frac{\rho_{H_a}}{0.003 * \rho_{H_g}}}$$

Estimating the Hall coefficient of the adsorbate phase at the highest observed carrier concentration (n=2E13cm^{-2}) by $\rho_{H_a} = \frac{1}{ne} = 312k\Omega$ and the graphene phase from the initial measurement $R_m = 300\Omega$ we obtain a sheet resistance of the adsorbate phase of $R_a > 200k\Omega/\square$.

Influence of graphene defectiveness

Defects in the graphene lattice are expected to affect both the carrier transport and adsorption process. In order to demonstrate the universality of the relation between morphology and doping, graphene with various degrees of defectiveness was produced by variation of the CVD growth process. Upon UV exposure all samples exhibit a decrease of mobility with increasing impurity

concentration as discussed in the manuscript. The charge transfer efficiency α quantifies the relation between both quantities. We find that all values for α are within an error of 8% which suggests that the graphene defectiveness does not affect the morphology and charge transfer behavior of adsorbates.

	Sample 1	Sample 2	Sample 3	Sample 4	Sample 5
I_D/I_G ratio	0.16	0.11	1.24	0.33	0.30
α	0.34	0.36	0.36	0.31	0.39

Table S5. Raman ID/IG ratio and α for 5 samples measured

Morphology of $AuCl_3$ adsorbates

The change of adsorbate morphology upon extended exposure was confirmed by characterization of $AuCl_3$ on graphene using AFM. It can be seen that the density and extent of the AuCl3 clusters is varying with exposure in agreement with previous reorts.[10] The finding confirms that the highest conductivity is achieved if large non-interacting clusters are formed (Figure S6(c)) and further exposure leads to decreased conductivity.

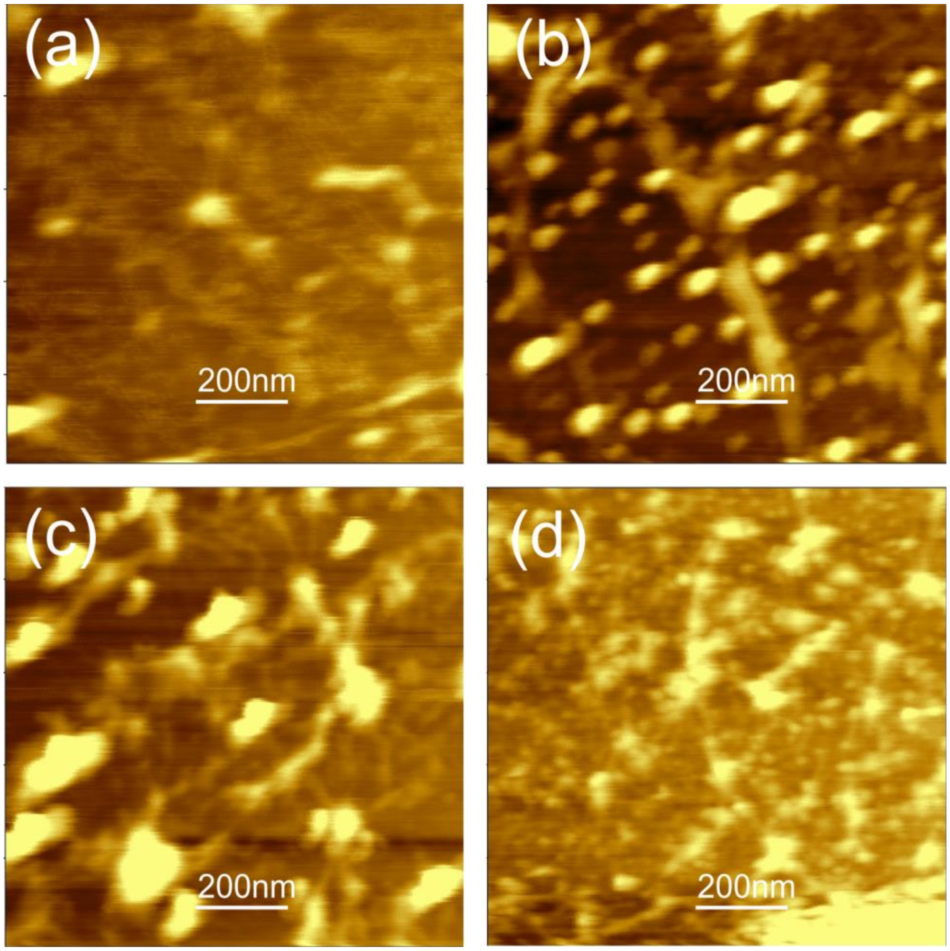

Figure S6. AFM images of graphene after varying exposure to AuCl3. (a) pristine graphene ($R_s = 2k\Omega/\square$), (b)short exposure ($R_s = 1k\Omega/\square$), (c)medium exposure ($R_s = 700\Omega/\square$), (d) long exposure ($R_s = 900\Omega/\square$)

References

1. Lee, G.; Lee, B.; Kim, J.; Cho, K., Ozone Adsorption on Graphene: Ab Initio Study and Experimental Validation. *The Journal of Physical Chemistry C* **2009,** *113* (32), 14225-14229.
2. Cançado, L. G.; Jorio, a.; Ferreira, E. H. M.; Stavale, F.; Achete, C. a.; Capaz, R. B.; Moutinho, M. V. O.; Lombardo, a.; Kulmala, T. S.; Ferrari, a. C., Quantifying defects in graphene via Raman spectroscopy at different excitation energies. *Nano letters* **2011,** *11* (8), 3190-6.

3. Tao, H.; Moser, J.; Alzina, F.; Wang, Q.; Sotomayor-Torres, C. M., The Morphology of Graphene Sheets Treated in an Ozone Generator. *The Journal of Physical Chemistry C* **2011,** *115* (37), 18257-18260.
4. Lopez, V.; Sundaram, R. S.; Gomez-Navarro, C.; Olea, D.; Burghard, M.; Gomez-Herrero, J.; Zamora, F.; Kern, K., Chemical Vapor Deposition Repair of Graphene Oxide: A Route to Highly Conductive Graphene Monolayers. *Adv. Mater.* **2009,** *21* (46), 4683-+.
5. Voggu, R.; Das, B.; Rout, C. S.; Rao, C., Effects of charge transfer interaction of graphene with electron donor and acceptor molecules examined using Raman spectroscopy and cognate techniques. *Journal of Physics: Condensed Matter* **2008,** *20* (47), 472204.
6. Yan, J.-A.; Chou, M. Y., Oxidation functional groups on graphene: Structural and electronic properties. *Physical Review B* **2010,** *82* (12), 125403-125403.
7. Lilliu, S.; Maragliano, C.; Hampton, M.; Elliott, M.; Stefancich, M.; Chiesa, M.; Dahlem, M. S.; Macdonald, J. E., EFM data mapped into 2D images of tip-sample contact potential difference and capacitance second derivative. *Scientific reports* **2013,** *3*, 3352-3352.
8. Klier, K., Theorie der randschicht fur einen begrenzten krystall des adsorbens. **1961**, 920-927.
9. Shin, H.-j.; Choi, W. M.; Choi, D.; Han, G. H.; Yoon, S.-m.; Park, H.-k.; Kim, S.-w.; Jin, Y. W.; Lee, S. Y.; Kim, J. M.; Choi, J.-y.; Lee, Y. H., Control of Electronic Structure of Graphene by Various Dopants and Their Effects on a Nanogenerator. **2010,** (16), 15603-15609.
10. Shi, Y.; Kim, K. K.; Reina, A.; Hofmann, M.; Li, L.-j.; Kong, J., Work Function Engineering of Graphene Electrode via Chemical Doping. **2010,** *4* (5), 2689-2694.
11. Iqbal, M. Z.; Siddique, S.; Iqbal, M. W.; Eom, J., Formation of p–n junction with stable p-doping in graphene field effect transistors using deep UV irradiation. *Journal of Materials Chemistry C* **2013,** *1* (18), 3078-3078.
